# The role of EGFR-TKI for leptomeningeal metastases from non-small cell lung cancer

**DOI:** 10.1186/s40064-016-2873-2

**Published:** 2016-08-03

**Authors:** Xu Yufen, Song Binbin, Chen Wenyu, Liu Jialiang, Yang Xinmei

**Affiliations:** 1 School of Medicine, Jiaxing University, Jiaxing, 314000 Zhejiang People’s Republic of China; 2 Department of Oncology, The First Affiliated Hospital of Jiaxing University, No. 1882, Zhonghuan South Road, Jiaxing, 314000 Zhejiang People’s Republic of China

**Keywords:** Non-small cell lung cancer, Leptomeningeal metastasis, EGFR-TKIs

## Abstract

Leptomeningeal metastasis (LM) is a terminal event in the development of non-small cell lung cancer (NSCLC). It has a poor prognosis with median survival of 1.9 months if untreated. The improvement of OS in NSCLC patients relatively increases incidence of LM. While current therapeutic options for LM are limited. Epidermal growth factor receptor-tyrosine kinase inhibitors are a class of small molecules and show dramatic response in epidermal growth factor receptor mutated patients. It also has a distinct therapeutic potential against brain metastases. Although there are some studies on EGFR-TKIs and brain metastases, the role of EGFR-TKIs on LM are not fully clarified. In this review, we will summarize current evidences concerning the use and discuss the role of EGFR-TKIs on LM.

## Background

Lung cancer is the most common cancer and leading cause of cancer deaths in the world (Ferlay et al. 2010). As improvement in treatment for non-small-cell lung carcinoma (NSCLC), previous rare complications become evident. Leptomeningeal metastasis (LM), namely cancer cells spreading to the meninges, is a terminal event in the development of NSCLC (Chamberlain 2005). LM occurs in 5 % of cancers, and 14–29 % of them derives from lung cancer (Grossman and Krabak 1999). LM has a poor prognosis with median survival of 1.9 months if untreated (Herrlinger et al. 2004). Intravenous chemotherapy is believed to have a limited role because its inability to form high drug concentrations in intracranial area due to blood brain barrier (BBB). Current available drugs for intrathecal chemotherapy (ITC) including methotrexate and cytarabine have limited antitumor activity in lung cancer (Oechsle et al. 2010). Although whole brain radiotherapy (WBRT) is an effective therapy for brain parenchyma metastasis, it does not exhibit survival benefit on LM (Morris et al. 2012). Epidermal growth factor receptor-tyrosine kinase inhibitors (EGFR-TKIs) are a class of small molecules and have demonstrated dramatic response rates from 60 to 90 % in sensitizing EGFR mutation NSCLC population as systemic therapy (Maemondo et al. 2010; Mitsudomi et al. 2010; Mok et al. 2009). EGFR-TKIs also showed a distinct therapeutic potential against brain metastases of NSCLC (Namba et al. 2004; Hotta et al. 2004), suggesting its ability to penetrate BBB into cerebrospinal fluid (CSF). In addition, EGFR-TKIs have antitumor efficacy in central nervous system metastases even for those patients with extracranial lesions failure after standard dose EGFR-TKIs treatment (Grommes et al. 2011; Jackman et al. 2006; Dhruva and Socinski 2009; Clarke et al. 2010; Kuiper and Smit 2013; Hata et al. 2011; Kawamura et al. 2015). Notwithstanding there are some studies on EGFR-TKIs and brain metastases, the role of EGFR-TKIs on LM are not fully clarified. In this review, we will summarize current evidences concerning the use and discuss the role of EGFR-TKIs on LM.

## Definition and diagnosis of LM

LM is defined as the spread of malignant cells to the leptomeninges and subarachnoid space and dissemination of tumors cells with the CSF compartment (Gleissner and Chamberlain 2006). Symptoms of LM contains mental changes, headaches, seizures, and back pain, etc. However, it can also exhibit some abscopal symptoms. Sim et al. reported a case chest wall pain as the presenting symptom of LM (Sim et al. 2014). The gold standard of diagnosis with LM is detection of malignant cells in CSF (Chamberlain 2005). While CSF examination can only perform in part of patients. Therefore, the finding of meningeal enhancement in cranial and/or spinal MRI combine with neurological symptoms are also recognized as LM.

## Current therapeutic modalities of EGFR-TKIs for LM

EGFR-TKIs open up era of target therapy on NSCLC and markedly prolong OS and PFS on EGFR_MT_ patients. The improvement of OS in NSCLC patients relatively increases incidence of LM. In the following part, we will focus on therapeutic options of EGFR-TKIs on LM. Table 1 summaries current clinical trials on application EGFR-TKIs for LM.

**Table 1:** Current clinical trials on EGFR-TKIs for LM

Author	Years	N	Study type	Regimen	VP	Histology	Prior EGFR-TKIs	Outcomes	References
Yang	2015	6	Retrospective	Standard-dose erlotinib + PP	No	EGFR_MT_ NSCLC	Gefitinib	Intracranial CR 1/6, PR 3/6; neurological symptoms improved 5/5, PS improved 6/6; median PFS 8.5 months; median OS 9.0 months	Yang et al. (2015)
Kawamura	2015	12	Retrospective	High-dose erlotinib ± WBRT/Bevacizumab	No	EGFR_MT_ NSCLC	Gefitinib/erlotinib	Intracranial ORR 3/10; neurological symptoms improved 6/12, PS improved 4/12; median PFS 2.3 months; median OS 6.2 months	Kawamura et al. (2015)
Jackman	2015	7	Phase I	High-dose gefitinib	No	EGFR_MT_ NSCLC	Gefitinib/erlotinib/Vandetanib	Intracranial ORR 0/7; neurological symptoms improved 4/7; median PFS 2.3 months; median OS 3.5 months	Jackman et al. (2015)
Gong	2015	21	Retrospective	High-dose/Standard-dose Icotinib ± ITC/WBRT/Chemotherapy	No	EGFR_MT_ NSCLC	Icotinib partly	Intracranial ORR 8/21; neurological symptoms improved 18/20, PS improved 17/21; median OS 10.1 months	Gong et al. (2015)
Lin	2014	1	Case report	Afatinib + cetuximab	No	EGFR_MT_ ADC	High-dose erlotinib, gefitinib	Intracranial PR; neurological symptoms improved; OS 5 months	Lin et al. (2014)
Kwon	2014	37	Retrospective	EGFR-TKIs	No	NSCLC	EGFR-TKIs partly	Median OS 10.5 months versus 3.0 months with or without EGFR-TKIs therapy after LM	Kwon and Chie (2014)
Lee	2013	25	Retrospective	Gefitinib/erlotinib ± ITC/WBRT	No	NSCLC	Gefitinib/erlotinib	Intracranial ORR 5/9 versus 3/9, median OS 9.5 months versus 4.4 months with erlotinib or gefitinib therapy after LM	Lee et al. (2013)
Kuiper	2013	2	Case reports	High-dose pulsatile erlotinib + Chemotherapy	No	EGFR_MT_ NSCLC	Erlotinib/afatinib	Intracranial PR 2/2; neurological symptoms improved 2/2	Kuiper and Smit (2013)
Yuan	2012	1	Case report	High-dose gefitinib + Pemetrexed	No	EGFR_MT_ ADC	Gefitinib	Intracranial PFS 6.0 months, neurological symptoms improved	Yuan et al. (2012)
Umemura	2012	91	Retrospective	Gefitinib/erlotinib ± ITC/WBRT	No	NSCLC	–	Median OS 5.3 months versus 2.3 months with or without EGFR-TKIs therapy after LM	Umemura et al. (2012)
Togashi	2012	1	Case report	Erlotinib	No	EGFR_MT_ ADC	Erlotinib/gefitinib	Intracranial PR; neurological symptoms improved; PS improved; PFS 8.0 months	Togashi et al. (2012)
Park	2012	50	Retrospective	Erlotinib/gefitinib ± WBRT	No	NSCLC	EGFR-TKIs partly	Median OS 4.3 months for all patients, median OS was longer with patients receiving EGFR-TKIs than not (*p* = 0.002)	Park et al. (2012)
Masuda	2011	3	Case reports	Erlotinib	No	EGFR_MT_ ADC	Gefitinib	Neurological symptoms improved 2/3; PS improved 2/3; median OS 93 days	Masuda et al. (2011)
Grommes	2011	9	Retrospective	High-dose pulsatile erlotinib	No	EGFR_MT_ NSCLC	Erlotinib/Afatinib/gefitinib	Intracranial ORR 6/9; neurological symptoms improved 18/20,; median PFS 2.7 months; median OS 12.0 months	Grommes et al. (2011)
Clarke	2010	1	Case report	High-dose pulsatile erlotinib	Yes	EGFR_MT_ NSCLC	Erlotinib	Intracranial PR; OS 14 months	Clarke et al. (2010)
Yi	2009	11	Retrospective	Erlotinib/gefitinib ± ITC	No	NSCLC	Gefitinib partly	Clinical response 9/11; >6 months OS 8/11	Yi et al. (2009)
Katayama	2009	4	Retrospective	Erlotinib	No	EGFR_MT_ ADC	Gefitinib	Intracranial ORR 2/3; neurological symptoms improved 3/4, PS improved 1/4; median OS 4.0 months	Katayama et al. (2009)
Dhruva	2009	1	Case report	High-dose pulsatile erlotinib	No	NSCLC	Erlotinib	Intracranial PR; neurological symptoms improved, PS improved	Dhruva and Socinski (2009)
Jackman	2006	1	Case report	High-dose gefitinib	No	EGFR_MT_ ADC	Gefitinib	Intracranial PR; neurological symptoms improved, PS improved	(Jackman et al. 2006)
Choong	2006	1	Case report	Gefitinib	No	EGFR_MT_ ADC	Erlotinib	Intracranial PR; neurological symptoms improved, PS improved	(Choong et al. 2006)

*N* total number, *VP* ventriculoperitoneal shunt, *EGFR*-*TKIs* epidermal growth factor receptor-tyrosine kinase inhibitors, *Ref* citation references, *PP* pemetrexed plus cisplatin, *WBRT* whole-brain radiotherapy, *ITC* intrathecal chemotherapy, *EGFR*_*MT*_ epidermal growth factor receptor mutation type, *NSCLC* non-small cell lung cancer, *ADC* adenocarcinoma, *CR* complete response, *PR* partial response, *PS* performance status, *PFS* progression-free survival, *OS* overall survival, *ORR* overall response rate, *LM* leptomeningeal metastasis

### EGFR mutation versus wild type

EGFR mutation type (EGFR_MT_) NSCLC is an important subgroup of NSCLC, accounting for about 50 % in Asian and 10 % in Caucasian population (Hirsch and Bunn 2009). Mounting evidences have shown EGFR-TKIs improved overall response rate (ORR), progression-free survival (PFS) and/or overall survival (OS) with minimal toxicities in EGFR sensitive mutation patients (Maemondo et al. 2010; Mitsudomi et al. 2010; Rosell et al. 2012; Sequist et al. 2013; Zhou et al. 2011). In the case of LM, EGFR_MT_ type also contributed to longer OS and PFS as well as better performance status (PS). A retrospective study conducted by Umemura et al. showed the median survival time (MST) for all NSCLC patients with LM was 3.6 months, while MST in exon 19 deletion, exon 21 mutation, and wild type patients were 11.0, 7.1, and 1.4 months. Similar results were seen in PFS. The median time to progression (mTTP) for these three group were 7.8, 2.0, and 0.9 months (Umemura et al. 2012). Although EGFR-TKIs have an initial good response, it inevitably moves toward resistance around 1-year (Maemondo et al. 2010; Mitsudomi et al. 2010; Mok et al. 2009). Several mechanisms lead to EGFR-TKIs resistance, including secondary mutation (Primary T790M mutation), c-Met amplification, and transformation to small cell lung cancer (Kobayashi et al. 2005). T790M mutation is an important mechanism of acquired resistance, and there are about 50 % T790M mutation in patients after TKI failure (Sequist et al. 2011; Oxnard et al. 2011; Chen et al. 2009; Balak et al. 2006). Most of LM occurs at late course of NSCLC, patients usually have acquire resistance of EGFR-TKIs. However, intracranial metastases retain sensitive mutation even when extracranial lesions develop secondary mutation such as T790M (Jackman et al. 2006; Clarke et al. 2010; Balak et al. 2006; Heon et al. 2010). Lack of selection pressure as poor penetration of TKIs in intracranial metastases might explain this phenomenon.

### First, second, and third-generation EGFR-TKIs

As first-generation EGFR-TKIs, erlotinib (Tarceva) and gefitinib (Iressa) exhibit dramatic efficacy in selected NSCLC patients. Each of them has evidence proving their efficacy as first-line, second-line, third-line, or maintenance therapy (Mok et al. 2009; Qi et al. 2012; Alimujiang et al. 2013). Certainly, there are some studies comparing efficacy and toxicity between erlotinib and gefitinib. Burotto et al. found both of them had similar toxicity profiles as well as outcomes including ORR, PFS, and OS (Burotto et al. 2015). However, Wu et al. conducted a study enrolled 716 patients, and found different effectiveness between gefitinib and erlotinib for NSCLC_MT_ patients (Wu et al. 2011). In addition, several studies showed erlotinib produced clinical benefits in some patients after gefitinib failure (Hata et al. 2011; Kaira et al. 2010). Similar phenomenon was seen in LM. Several studies reported erlotinib had a good response for LM after gefitinib failure (Yang et al. 2015; Yuan et al. 2012; Masuda et al. 2011; Katayama et al. 2009). Erlotinib was believed to have more penetration rate into the CSF than gefitinib. Togashi et al. compared CSF concentration and penetration rate between gefitinib and erlotinib in 15 patients (Togashi et al. 2012). The results showed the CSF concentration for gefitinib and erlotinib were 8.2 ± 4.3 nM and 66.9 ± 39.0 nM, respectively. The penetration rate were 1.13 ± 0.36 and 2.77 ± 0.45 %, respectively. Patients with erlotinib also achieved a preferentially higher intracranial response rate than those with gefitinib (4/7 vs. 1/3). Another study retrospectively analyzed the two EGFR-TKIs efficacy on 25 NSCLC patients with LM (Lee et al. 2013). They found patients with erlotinib showed better cytological conversion rate of LM than those with gefitinib (9/14 vs. 1/11). In addition, another first-generation EGFR-TKI icotinib also showed efficacy on LM from NSCLC with EGFR mutation (Gong et al. 2015). Of course, second-generation (Afatinib) and third-generation TKIs (AZD9291) have been applied in clinical practice or clinical trials. Lux-Lung 3 and Lux-Lung 6 showed first-line afatinib significantly prolonged PFS compared to platinum-based chemotherapy (Sequist et al. 2013; Wu et al. 2014). Pooled analysis of Lux-Lung 3 and Lux-Lung 6 showed afatinib improved OS in patients with del19 EGFR mutations but not Leu858Arg EGFR mutations, indicating the potential mechanistic differences between afatinib and first-generation EGFR-TKIs (Yang et al. 2015). Recent two studies Lux-Lung 7 and Lux-Lung 8 compared afatinib with gefitinib and erlotinib showing superiority of afatinib in PFS and OS (Park et al. 2016; Soria et al. 2015). Lin et al. reported a case afatinib combination with cetuximab was effective for LM after erlotinib/gefitinib failure (Lin et al. 2014). Another report on outcomes of pretreated NSCLC patients with CNS metastasis who received afatinib showed that thirty-five percent (11 of 31) of evaluable patients had a cerebral response (Hoffknecht et al. 2015). CSF data from one patient showed afatinib CSF concentration of approximately 1.0 nM. Afatinib appears to penetrate into the CNS with concentrations high enough to have clinical effect on CNS metastases. Osimertinib (AZD9291) is a third-generation EGFR-TKI. BLOOM study showed osimertinib demonstrated encouraging preliminary activity in heavily pretreated patients with LM disease from EGFR^MT^ NSCLC (Yang 2016). Ahn et al. analyzed patients with brain metastasis from AURA and AURA2 studies and showed osimertinib achieved an ORR of 56 % (Ahn et al. 2015). CSF data from one patient showed AZD9291 CSF concentration of 3.44 nM. Nanjo et al. established an in vivo LM mice model and found osimertinib markedly inhibited progression of LM even after refractory to erlotinib (Nanjo et al. 2016).

### High-dose versus standard dose EGFR-TKIs

Traditional chemotherapy is unable to penetrate into CSF due to BBB. This causes limited efficacy of systemic therapy on intracranial lesions. EGFR-TKIs are a class of small molecular agents and supposed to easily penetrate into the CSF, however, the penetration rate is still low. Therefore, intracranial failure of EGFR-TKIs is consider as pharmacokinetic failure. Higher dose EGFR-TKIs is supposed to correlate with higher concentrations in the CSF. Togashi et al. studied the relationship between plasma and CSF concentrations of erlotinib (Togashi et al. 2011). The authors found the plasma erlotinib concentrations at a dose of 75 and 150 mg were 433 and 1117 nM, corresponding to CSF concentrations 14 and 44 nM. There was a good correlation between plasma and CSF concentrations (R^2^ = 0.84, *p* < 0.001). For that matter, a series studies have evaluated the efficacy of high-dose EGFR-TKIs on intracranial metastases from NSCLC. Kawamura et al. analyzed safety and efficacy of 35 patients with or without high-dose erlotinib and found high-dose erlotinib significantly improved PS and neurological symptoms with no elevated rates of severe adverse events (AEs) as compared to standard-dose EGFR-TKIs (Kawamura et al. 2015). Median OS in high-dose erlotinib and standard-dose EGFR-TKIs were 6.2 and 5.9 months, respectively (Kawamura et al. 2015). Jackman et al. conducted a phase I study of high-dose gefitinib on LM from NSCLC and found high-dose gefitinib could improve neurological symptoms with minor toxicity (Jackman et al. 2015). Several studies reported high-dose EGFR-TKIs improved neurological symptoms and decreased intracranial metastases after standard-dose EGFR-TKIs failure (Dhruva and Socinski 2009; Clarke et al. 2010; Kuiper and Smit 2013; Hata et al. 2011; Yuan et al. 2012).

### Combination with WBRT

WBRT has an important role in brain metastases, while its impact on LM is unknown. A study of Morris et al. showed WBRT might play a role in symptom control, but it did not improve OS of LM patients (Morris et al. 2012). Although WBRT alone does not exhibit superior outcomes, combination with EGFR-TKIs seems to be effective. In Yang’s study, four patients achieve partial or complete response who received WBRT, while the other two patients achieve stable disease who did not combine with WBRT (Yang et al. 2015). Of course, the sample size is small, further study are needed to evaluate WBRT combination with EGFR-TKIs.

## Conclusion

LM is a terminal event of NSCLC and its prognosis is extremely poor. Current therapeutic options for LM are limited. EGFR-TKIs as important target drugs exhibit good efficacy for EGFR_MT_ patients with LM. In this review, we discuss selection and therapeutic strategy of EGFR-TKIs for LM. EGFR_MT_ patients with LM might be more appropriate for EGFR-TKIs therapy than those EGFR wild type patients. It seems erlotinib and high-dose regimen could benefit more patients. Also combination with WBRT is another option. Nevertheless, current evidence are derived from respective and case reports study, further randomized control trials are needed to fully understand the role of EGFR-TKIs for LM.

## Abbreviations

ADC: adenocarcinoma
AEs: adverse events
BBB: blood brain barrier
CR: complete response
CSF: cerebrospinal fluid
EGFR: epidermal growth factor receptor
EGFRMT: EGFR mutation type
ITC: intrathecal chemotherapy
LM: leptomeningeal metastasis
MST: median survival time
mTTP: median time to progression
NSCLC: non-small cell lung cancer
ORR: overall response rate
OS: overall survival
PFS: progression-free survival
PR: partial response
PS: performance score
TKI: tyrosine kinase inhibitor
VP: ventriculoperitoneal shunt
WBRT: whole brain radiotherapy
